# The contribution of alliaceous and cruciferous vegetables to dietary sulphur intake

**DOI:** 10.1016/j.foodchem.2017.04.098

**Published:** 2017-11-01

**Authors:** Joanne F. Doleman, Katrijn Grisar, Lena Van Liedekerke, Shikha Saha, Mark Roe, Henri S. Tapp, Richard F. Mithen

**Affiliations:** a Food & Health Programme, Institute of Food Research, Norwich, UK; b Food Databanks, Institute of Food Research, Norwich NR4 7UA, UK; c Analytical Sciences Unit, Institute of Food Research, Norwich NR4 7UA, UK

**Keywords:** Sulphur, Sulphur amino acids (SAA), Sulphate, Diet diary, Duplicate diet

## Abstract

Despite its importance in many areas of human metabolism, there are no recommended daily intake guide lines for sulphur. It is generally assumed that most dietary sulphur originates from intake of methionine and cysteine. We estimated sulphur intake from food diaries, and validated the results with the use of a duplicate diet analyses. Sulphur intake estimations were highly correlated with that obtain through an elemental analysis of duplicate diets, with a mean ± sd daily intakes of 956 ± 327.9 mg estimated from diet diary analyses and 935 ± 329.9 mg estimated by a duplicate diet analyses. Sulphur intake from alliaceous and cruciferous vegetables contributed up to 42% of total sulphur intake. Daily intake estimation comparisons through diet diary analyses and duplicate diet for other elements showed good agreement, except for sodium and zinc, in which analyses of 24 h diet dairies overestimated intake by 35% and 52%, respectively.

## Highlights

• Sulphur intake estimation based upon methionine and cysteine overlooks contributions from other sources.
• Intake estimations for sulphur from diet diary and duplicate diet agree well.
• There is no requirement for mass re-analysis of the sulphur content of foods.
• Caution is advised for diet diary analysis estimations of zinc and sodium.

## 1 Introduction

It is generally considered that most dietary sulphur is derived from the sulphur-containing amino acids (SAA), methionine and cysteine, with other forms of organic sulphur, such as the specialised metabolites that accumulate in alliaceous and cruciferous vegetables contributing a very small percentage to total sulphur intake. While there are recommended daily allowance (RDA) for SAA, based upon the requirements to maintain nitrogen balance, there is not a RDA or an ‘adequate intake estimation’ for sulphur, despite its importance in many aspects of metabolism (Nimni, Han, & Cordoba, 2007). This lack of recommended intakes is reflected in current methods of dietary analyses that do not provide an estimate of sulphur intake, and do not attempt to identified important sources of sulphur in the diet other than SAA.

The likely major sources of sulphur in the diet other than SAA are inorganic sulphate in drinking water and foods, and the sulphur-containing specialised metabolites that are characteristic of *Allium* vegetables, such as onions, leeks and garlic, and cruciferous vegetables, such as cabbages, kales and broccoli. The levels of sulphate in tap water varies around the world, with low concentrations measured in tap water from the Netherlands (below 260 µ/L) and higher levels in central Canada (up to 22 mmol/L). A water quality survey of British tap water suppliers measured a mean sulphate concentration of 620 µmol/L, and a maximum of 2458 µmol/L (Powell, Bailey, & Jolly, 1987). Sulphate ingestion has been of interest due to the potential importance of sulphate reducing bacteria (SRB) in the colon, and their association with ulcerative colitis, irritable bowel disease, and Crohn’s disease (Salonen, de Vos, & Palva, 2010), and inorganic sulphur is required for synthesis of 3′-phosphoadenosine-5′-phosphosulfate required for detoxification of endogenous metabolites and xenobiotics. The consumption of both alliaceous and cruciferous vegetables have been associated with health benefits (McNaughton and Marks, 2003, Nicastro et al., 2015), including a reduced risk of developing various cancers including breast, prostate, lung, pancreatic and gastrointestinal (Herr & Buchler, 2010), cardio vascular disease (Zhang et al., 2011), reduced risk of type-2 diabetes (Kurotani et al., 2013), and protection from neurodegenerative disease (Giacoppo et al., 2015). For cruciferous vegetables, these health benefits are associated with the consumption of glucosinolates, the sulphur-rich compounds that accumulate in cruciferous vegetables, and their metabolic derivatives (Armah et al., 2015; Maheo et al., 1997; Yamagishi & Matsui, 2016). Likewise, for alliaceous vegetables, these putative health benefits are attributed to S-2-propenyl-l-cysteine S-oxide and other related cysteine S-oxides (Block, 1992) which have been shown to inhibit cell proliferation and induce cell death of cancerous cells (Borlinghaus, Albrecht, Gruhlke, Nwachukwu, & Slusarenko, 2014).

In this paper, we provide a method for evaluating sulphur intake based upon food diary analyses and validate this with the use of a duplicate diary analyses. Subsequently, we provide an estimation of the proportion of dietary sulphur that comes from non-SAA sources. The study was extended further to look at daily intakes for other commonly consumed minerals including sodium, calcium, iron, zinc, magnesium, phosphorous and potassium and the validity of current intake estimations from diet diary analysis.

## 2 Materials & methods

### 2.1 Analysis of commonly consumed foods for sulphur content

Thirty-two commonly consumed foods were purchased for sulphur analysis including fruits and vegetables, meat, fish, pasta, rice and eggs. Fruits and vegetables were analysed raw and included spring onions, red onions, brown onions, onions, leek, garlic, parsnip, potato, sweet potato, green pepper, courgette, carrot, tomato, sugar snap peas, dwarf beans, Braeburn apple, lettuce, wild rocket, radish, savoy cabbage, sweetheart cabbage, curly leaf kale, broccoli and cauliflower (frozen). Cooked ham, plain flour, white bread and minced beef (20% fat) were analysed as purchased. Chicken breast fillets and boneless cod fillets were pan fried without fat prior to analysis. Long grain rice, spaghetti pasta and medium free range eggs were boiled prior to analysis. All samples were weighed, freeze dried and weighed again prior to grinding to a fine powder. Freeze dried samples were sent to Eurofins UK (Eurofins Food Testing UK Ltd, i54 Business Park, Valiant Way, Wolverhampton, WV9 5 GB) for analysis of amino acids and minerals, in which the sulphur, sodium, potassium, calcium, magnesium, phosphorus, iron and zinc content of each sample was measured. Eighteen of the vegetable samples were also analysed in-house using established methods for glucosinolate content (Gasper et al., 2005).

For analysis of sulphate, 25 mg PVPP (polyvinylpolypirrolidone; washed and ground) was soaked in 1 ml H_2_O, overnight at 4 °C. 20–30 mg ground, frozen sample was added and mixed and shaken for 1 h at 4 °C. The sample was incubated for 15 min at 95 °C, centrifuged 15 min at 4 °C and supernatant filtered by PVDF syringe filter into an HPLC vial for analysis by LC-MS with a Agilent 1100 HPLC system and a mass spectroscopy detector (Agilent Technologies, Waldbronn, Germany). Samples were eluted at 0.5 ml/min with a gradient mobile phase. Mobile Phase A was 200 mM ammonium acetate buffer at pH 4.0 and B was 60% acetonitrile. The gradient started at 5% solution A increasing over 15 min to 90% A and finally re-equilibrated to 5% A for 5 min. Sulphate was monitored using mass spectrometry in selective ion monitor mode (*m*/*z* = 97) in negative polarity with electrospray ionization. The quantification was performed using matrix match calibration curve.

### 2.2 Sulphur database construction

An on-line literature search was conducted to identify sources of published food composition data for the sulphur content in foods. One key paper, two databases and five book supplements were identified that contained sulphur content information and these were imported into our own sulphur database containing 1222 different foods from the different reference sources (FoodStandardsAgency, 2008, Gnagnarella and Salvini, 2008, Holland et al., 1988, Holland et al., 1991, Holland et al., 1992, Holland et al., 1989, Masters and McCance, 1939, PublicHealthEngland, 2015). To aid navigation through the database, the foods were grouped into the following eleven categories: vegetables, fruits and nuts, cereals, meat, fish, dairy, beverages, condiments, fats, cooked dishes and miscellaneous foods.

### 2.3 Computational analysis software

DietPlan6 (http://www.foresoft.co.uk) is a computer programme for the nutrition analysis of recipes, meals, menus and personal weighed-intake food diaries and contains approximately 5500 types of UK foods and allows other national food data tables to be imported and more foods to be added by the user. The database also includes Dietary Reference Values (Ashwell, 1991) Food Portion Sizes (MAFF, Ministry of Agriculture, & F. a. F, 1993) and Food Labelling Data (EU Regulation 1169/2011). For each of the different foods contained within dietary intervention diaries used in this study a new sulphur database code was developed that incorporated all the existing DietPlan6 nutritional information supplemented with the sulphur content from the newly developed database.

### 2.4 Validation of sulphur database

The consistency of the collected sulphur data was compared between the different data sources. Few differences were identified and where differences arose we chose the sulphur data to use in the database on a case by case basis. Where data was available from more than one source, the value used was a combination of the most up to date and/or the most frequently quoted data in the source documents. Recipes from a previous publication (Masters & McCance, 1939) were compared to recent recipes to ensure they were still produced using the same kinds of ingredients to maintain validity of the historic sulphur data prior to inclusion in the database.

### 2.5 Diet diaries

Diet diaries from the BASH human dietary intervention study (rec ref 12/EE/0313 clinical trial Number NCT01929564), which was carried out in collaboration between the Institute of Food Research and University of Reading, were used for dietary analysis. Detailed instructions describing how to complete the diet diary were provided, including photographs of food portion sizes and drinking vessels and their volumes, as well as details of how to record both processed meals and homemade recipes. Participants were instructed to record the details of all meals, snacks and drinks consumed, estimating or measuring the quantity of each item and the time of consumption. Participants were encouraged to be as accurate as possible when completing the dairies and were provided with an example completed diary.

In total, 41 one-day diet diaries from 32 different individuals were coded for the computational analysis of calories and nutrients and sulphur content in DietPlan6 software using the sulphur database codes developed specifically for this analysis. Dishes, recipes and commercial food were broken down into their individual ingredients. Daily sulphur intake was estimated and used to identify, low, moderate and high consumers of sulphur.

### 2.6 Duplicate diet analysis

Eighteen diet diaries encompassing low, moderate and higher consumers of sulphur, as identified from the initial 41 diet diaries, were selected. The recorded food items were purchased from local supermarkets and stored as appropriate, until required. Using the exact cooking methods and quantities consumed, the total food and drink intake for the 24-h period was recreated and blended to produce a homogeneous sample to represent each diet diary. Diet samples were stored at −20 °C prior to inorganic and moisture content analysis by Eurofins Food Testing Laboratory (Wolverhampton, UK).

### 2.7 Statistical analysis

Statistical analyses were performed using Minitab version 17.2.1, and Matlab (version 8.5), with the Statistics and Machine Learning Toolbox (version 10.0). Differences between duplicate diet analysis and DietPlan6 estimations were compared using paired t-tests and presented using Bland Altman plots (Bland & Altman, 1986). Linear trends in the differences were assessed from the significance of the Pearson correlation between the sum and difference of the two estimates.

## 3 Results and discussion

### 3.1 Sulphur partitioning in commonly consumed foods

Thirty-two commonly consumed foods were analysed for total sulphur content and the proportion of sulphur from SAA, and a subset of 18 vegetables were also analysed for the contribution of sulphur from glucosinolates and sulphate (Table 1). As expected the proportion of SAA was very high in fish, chicken and minced beef (74–97%). SAA are also the predominant source of sulphur compounds in rice, pasta and eggs. In alliaceous vegetables, a low proportion of the total sulphur in the vegetables was in the form of SAA (e.g. garlic 10.5%, red onions 10.5%), whereas for starchy vegetables the proportion of total sulphur in the form of SAA was much higher (e.g. sweet potato 80.8%, parsnip 62.7%). In wild rocket, radish, broccoli, sweetheart cabbage and curly leaf kale a proportion of the total sulphur content was from glucosinolates with the contribution ranging from 7.4% in curly leaf kale to 59.3% in radish. Sulphate contribution ranged from 1.0% (garlic) to 61.7% (carrot) of the total sulphur.

**Table 1:** Sulphur content in µmoles/g dry weight of 32 commonly consumed foods, portioned to show the proportion derived from the sulphur amino acids methionine and cysteine and other sulphur (including sulphate). The sulphur partitioning of sulphur containing metabolites of 18 commonly consumed vegetables including tryptophan and methionine derived glucosinolates, sulphur amino acids cysteine and methionine, sulphate and other sulphur is also included. (– indicates not measured).

	Sulphur amino acids Cysteine & Methionine µmoles/g dry weight (% of total sulphur)		Other sulphur including sulphate µmoles/g dry weight (% of total sulphur)		Sulphur partitioning of sulphur containing metabolites in 18 commonly consumed vegetables			
					Sulphate µmoles/g dry weight (%)	Met derived glucosinolate (µmoles/g dry weight)	Trp derived glucosinolate (µmoles/g dry weight)	Other sulphur excluding glucosinolates (µmoles/g dry weight)
Spring onion	23.6	(26.5)	65.3	(73.5)	17.1 (19.2)	0.0	0.0	48.2
Red onion	12.7	(10.5)	108.6	(89.5)	–	–	–	–
Brown onion	9.3	(12.0)	67.7	(88.0)	6.6 (8.6)	–	–	–
Leek	27.5	(22.7)	93.5	(77.3)	16.2 (13.4)	0.0	0.0	77.3
Garlic	26.5	(10.5)	225.8	(89.5)	2.4 (1.0)	0.0	0.0	223.4
Parsnip	12.8	(62.7)	7.6	(37.3)	10.6 (51.7)	0.0	0.0	0.0
Potato	20.9	(54.4)	17.5	(45.6)	13.0 (34.0)	0.0	0.0	4.5
Sweet potato	23.8	(80.8)	5.7	(19.2)	8.8 (29.8)	0.0	0.0	0.0
Green pepper	19.5	(34.4)	37.2	(65.6)	–	–	–	–
Courgette	43.1	(72.8)	16.1	(27.2)	11.7 (19.7)	0.0	0.0	4.4
Carrot	13.0	(42.1)	17.8	(57.9)	19.0 (61.7)	0.0	0.0	0.0
Tomato	25.1	(59.3)	17.3	(40.7)	13.6 (32.1)	0.0	0.0	3.7
Sugar snap peas	26.3	(44.7)	32.6	(55.3)	19.5 (33.0)	0.0	0.0	13.2
Dwarf beans	32.5	(61.4)	20.5	(38.6)	11.7 (22.1)	0.0	0.0	8.8
Lettuce	26.1	(51.0)	25.1	(49.0)	18.9 (37.0)	0.0	0.0	6.2
Wild rocket	82.4	(14.9)	469.1	(85.1)	319.9 (58.0)	126.6	1.8	149.2
Radish	17.8	(49.4)	18.3	(50.6)	27.0 (74.7)	19.6	33.2	0.0
Savoy cabbage	40.7	(21.6)	148.0	(78.4)	–	–	–	–
Cauliflower	44.3	(23.3)	145.6	(76.7)	–	–	–	–
Broccoli	56.1	(20.4)	218.5	(79.6)	87.7 (31.9)	24.2	10.3	130.8
Sweetheart cabbage	28.5	(28.9)	70.1	(71.1)	26.0 (26.4)	14.6	5.7	44.1
Curly leaf kale	49.4	(16.0)	259.5	(84.0)	196.7 (63.7)	17.9	6.8	62.8
Braeburn apple	3.8	(84.2)	0.7	(15.8)	–	–	–	–
Cooked ham	140.2	(80.0)	35.1	(20.0)	–	–	–	–
Minced beef	117.9	(74.7)	39.9	(25.3)	–	–	–	–
Chicken breast	247.6	(97.4)	6.6	(2.6)	–	–	–	–
Cod	288.8	(85.0)	51.1	(15.0)	–	–	–	–
Eggs	232.6	(96.8)	7.8	(3.2)	–	–	–	–
Long grain rice	31.2	(91.9)	2.8	(8.1)	–	–	–	–
Spaghetti	37.8	(88.5)	4.9	(11.5)	–	–	–	–
Plain flour	35.2	(91.8)	3.1	(8.2)	–	–	–	–
White bread	31.8	(45.0)	38.9	(55.0)	–	–	–	–

### 3.2 Sulphur database construction and estimating dietary sulphur intakes

The sulphur intake determined from analysis of each 24-h food diary analysed ranged from 390 mg to 1414 mg total sulphur consumed in a 24-h period, and enabled the identification of low (<750 mg/day), moderate (between 750 and 1250 mg/day) and high sulphur intake (>1250 mg/day) diets across the 41 diet dairies analysed.

### 3.3 Sources of sulphur in the diet

Diet diary analysis enabled us to identify the sources of sulphur in the diet. We categorised the sources into eight groups which were meat, fish, dairy, vegetables, fruits, wheat and starchy foods, dishes (e.g. prepared foods such as stock cubes); and miscellaneous (foods that did not easily fit into any of the other categories e.g. eggs, coffee, wine). All 41 diet diaries were analysed and the specific sources of sulphur in each diary determined. The total sulphur from meat and fish consumption contributed up to 458 mg (38.2%) and 884 mg (62.5%) respectively of total sulphur intake. Wheat and starchy foods contributed up to 594 mg (46.9%); dairy contributed up to 426 mg (35.3%) and vegetables contributed a maximum of 359 mg (37.9%) of the total sulphur intake (Table 2). We were particularly interested in the sulphur intake originating from cruciferous and alliaceous vegetables consumption and as such performed an additional analysis to investigate what proportion of the sulphur intake came from these vegetables. Cruciferous and alliaceous vegetables combined contributed up to 41.7% of the total sulphur intake. The highest contribution from cruciferous vegetables was 39.9% and the maximum contribution from alliaceous vegetables in any one diet diary analysed was 7.3% (Table 2). In 20 of the 41 diet diaries analysed cruciferous and alliaceous vegetable consumption represented more than 50% of the total sulphur consumed from all vegetables.

**Table 2:** Summary data table showing the mean sulphur intake (mg), % of total sulphur intake, intake range and the standard deviation across 8 food categories used in the analysis of 41 × 24-h diet diaries analysed using DietPlan6 with the sulphur database incorporated. The contribution of sulphur from the consumption of cruciferous and /or alliaceous vegetables is also included.

Food Category	Mean sulphur intake, mg	Mean % of total sulphur intake	Range mg (%)	Standard Deviation
Meat	142.1	15.8	0–458 (0–38.2)	135.5
Fish	93.1	10.4	0–884 (0–62.5)	200.5
Dairy	130.7	14.5	0–426 (0–35.3)	98.6
Vegetables	149.6	16.7	0–359 (0–37.9)	101.4
Fruits	22.2)	2.5	0–189 (0–18.6)	32.7
Wheat and starchy foods	152.8	17.0	0–584 (0–46.9)	119.1
Dishes	66.0	7.3	0–516 (0–62.0)	121.4
Other	141.9	15.8	4–559 (0.5–48.0)	125.9
Total sulphur consumed	898.3		361–1417	296.2
Cruciferous & alliaceous vegetables	67.3	7.5	0–335 (0–41.7)	79.9
Alliaceous vegetables	9.7	1.1	0–41 (0–7.3)	13.2
Cruciferous vegetables	57.6	6.4	0–320 (0–39.9)	78.2

### 3.4 Validity of the sulphur data in the historic databases

**Fig. 1:**
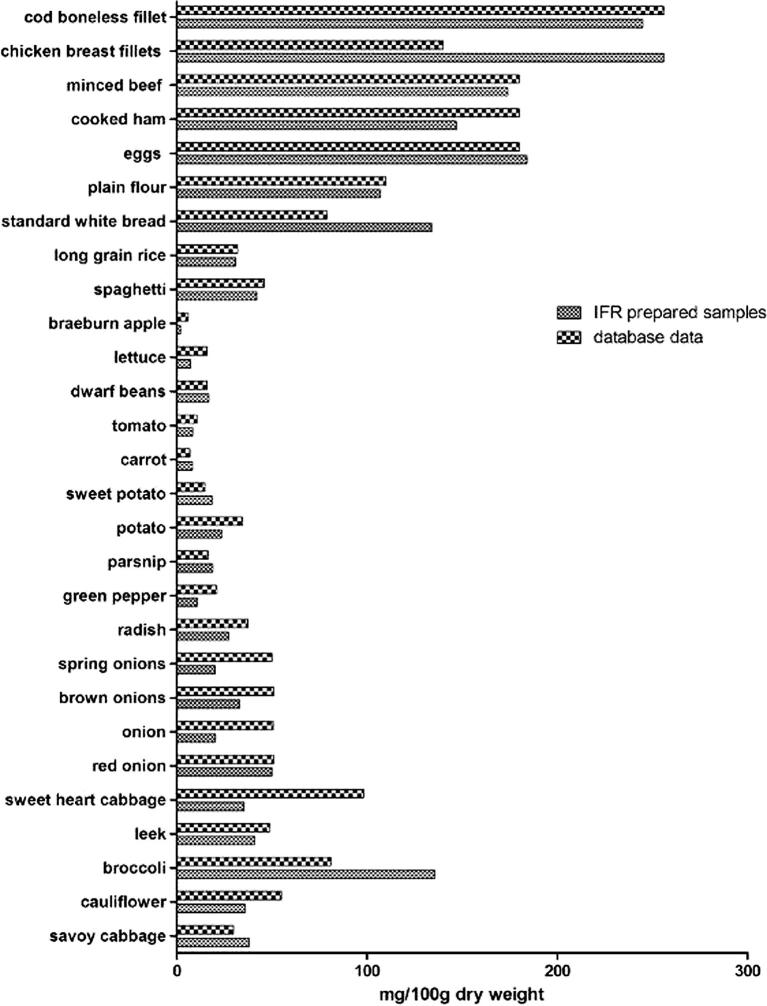
Sulphur content of 28 commonly consumed foods from the sulphur database versus Eurofins UK analysis of IFR in-house prepared samples. The sulphur content in mg per 100 g fresh weight of each food tested was comparable to that in the sulphur database, although some differences are observed. There does not appear to be any obvious systematic bias in the analysis.

Of the 32 different commonly consumed foods selected for sulphur analyses, including vegetables, fruit, meat, fish, pasta and rice, 28 were present in the sulphur database we constructed (absent were garlic, courgette, sugar snap peas and wild rocket). Much of the data collated in the sulphur database originated from the old components dataset of McCance and Widdowson’s ‘The Composition of Foods Integrated Dataset’ (PublicHealthEngland, 2015), the accuracy of this historical sulphur data was checked against the current sulphur content of various foods and vegetables by performing an in-house sulphur analysis of 32 commonly consumed foods. Samples were prepared and analysed as described in the materials and methods section for total sulphur content. The sulphur content of the analysed foods was compared to that in the sulphur database. In most cases, we could show good agreement between our in-house sulphur data and that in the collated sulphur database (Fig. 1).

Differences between the two sources of data were evident for onions, spring onions and sweetheart cabbage. The difference in the data for sweetheart cabbage was not unexpected as we were only able to obtain an average cabbage sulphur content and not a specific value for sweetheart cabbage. The data we obtained for the sulphur content of the onions and spring onion by our in-house analysis was lower than expected (1.9 and 2.85 mg/g dry weight respectively), and this was also lower than recently published data in which the sulphur content of 9 different onion varieties was tested and ranged from 3.5 to 5.1 mg/g dry weight (Lee, Yoo, Jifon, & Patil, 2009). The low sulphur content in the in-house analysis of onions and spring onions could be due to seasonal variation and the variety tested in this instance. The historic sulphur data for onions and spring onions, which aligned with the recently published data on the sulphur content of onions, was used in the sulphur database (Lee et al., 2009). Although some differences were observed, there did not appear to be any obvious systematic bias indicating that the sulphur data included in the database was representative of foods currently available to consumers.

### 3.5 Duplicate diet analysis

**Fig. 2:**
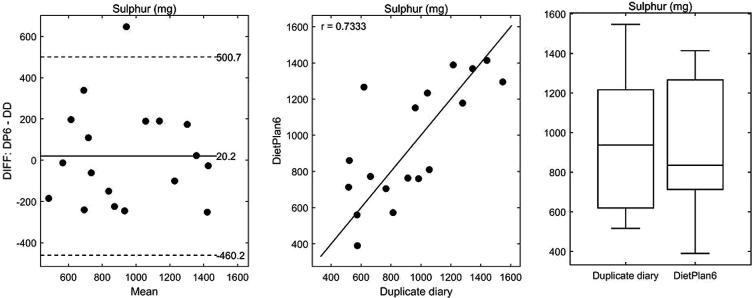
Sulphur intake estimates of 18 × 24-h dietary intake diaries using DietPlan6 and duplicate diet analysis. *Left panel*, Bland and Altman plot (Bland & Altman, 1986) of the differences, DietPlan6 – duplicate diary, against the mean of the two estimates. Also shown is the bias given as the mean difference and the 95% limits of agreement (mean difference ± 2 × standard deviation). *Central panel*, scatterplot of DietPlan6 estimates against duplicate diary values. Also shown is the Pearson correlation between the two methods and the line of unity. *Right panel*, boxplots summarising the distribution of the two estimates.

Duplicate diet analysis enabled us to assess the validity of the sulphur intake estimates compared to the historical sulphur data used in diet diary analysis. Of the 41 diet diaries analysed a subgroup of 18 diet diaries that encompassed 6 low, 6 moderate and 6 high sulphur intakes were analysed by duplicate diet analysis as described in the materials and methods section. These analytical data were compared to the sulphur intakes calculated from diet diary analysis using the Bland Altman method (Fig. 2). There was a strong positive linear association between the two methods (*r* = 0.73) and little evidence of a difference between the two methods (bias = 20.2 mg; *P* = 0.73; Table 3) or of any systematic trend in the differences against the mean (*P* = 0.97; Table 3). Total sulphur intake estimated by diet diary analysis across the 41 diet diaries ranged from 11.3–44.2 mmol/day and from the 18 duplicate diets the total dietary sulphur ranged from 16.1–48.3 mmol/day, thus showing little difference in the range of sulphur intake estimated between the two methods.

**Table 3:** Summary statistics for a selection of constituents estimated during the diet diary analysis by DietPlan6 and by duplicate diet analysis.

Constituent	*n*	DietPlan6		Duplicate diet		*DIFF: DP6-DD*			Trend
		Mean	±SD	Mean	±SD	Mean	±SD	*P diff*	*P trend*
Sulphur (mg)	18	956	±327.9	935	329.9	20.2	240.2	0.7251	0.9726
Sodium (mg)	18	2500	±1752	1764	1295	736	612	**0.0001**	**0.0003**
Calcium (mg)	18	890	±378.7	955	400.4	−65	299.8	0.3707	0.7576
Iron (mg)	18	14.33	±5.446	13.47	6.696	0.86	4.355	0.4129	0.2173
Magnesium (mg)	18	322.6	±96.65	339.2	98.44	−16.6	47.92	0.1610	0.8795
Phosphorus (mg)	18	1485	±460.1	1349	458.6	136	290.8	0.0625	0.9835
Potassium (mg)	18	3466	±1237	3785	1149	−319	758	0.0918	0.6297
Zinc (mg)	18	11.07	±4.724	8.36	3.110	2.71	2.791	**0.0007**	**0.0079**

*n*, sample size; *SD*, standard deviation; *DIFF: DP6-DD*, paired difference in the estimates by DietPlan6 and duplicated diary analysis; *P diff*, significance of difference based on paired *t*-test; *P trend*, significance of linear trend based on the correlation between the sum and difference of the two estimates; mg, milligram.

The total daily sulphur intake measured by both methods in this study is comparable to the daily sulphur intake estimations proposed by Florin and Ingenbleek et al. (Florin et al., 1991, Ingenbleek and Kimura, 2013). In other studies a moderately high total sulphur intake has been reported to be 38.2 mmol/day (high 42.2 mmol/day & very high 57.6 mmol/day) (Curno, Magee, Edmond, & Cummings, 2008). Using these figures a portion of the diet diaries in this study falls into the moderately high and high total sulphur intake category, however none of the diet diaries analysed could be classified as very high sulphur intake.

### 3.6 Analysis of sodium in the diet using duplicate diet and diet diary analyses

**Fig. 3:**
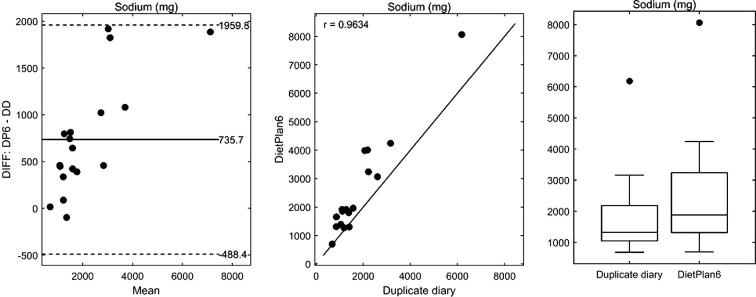
Sodium intake estimates of 18 × 24-h dietary intake diaries using DietPlan6 and duplicate diet analysis. *Left panel*, Bland and Altman plot (Bland & Altman, 1986) of the differences, DietPlan6 – duplicate diary, against the mean of the two estimates. Also shown is the bias given as the mean difference and the 95% limits of agreement (mean difference ± 2 × standard deviation). *Central panel*, scatterplot of DietPlan6 estimates against duplicate diary values. Also shown is the Pearson correlation between the two methods and the line of unity. *Right panel*, boxplots summarising the distribution of the two estimates.

As both the duplicate diet analysis and diet diary analysis provided information on other minerals we also investigated the agreement between the two methods for dietary sodium (Fig. 3) and various other minerals (Table 3). For sodium, there was a strong positive linear association between the two methods (*r* = 0.963). However, DietPlan6 showed a tendency to produce proportionately higher estimates as compared to duplicate diets (ratio of standard deviations, DP6/DD = 1.35, significance of linear trend, *P* = 0.0003). There was also strong evidence of a positive bias (mean difference = 736 mg, *P* = 0.0001). The dataset used for sodium content of foods in diet diary analysis was McCance and Widdowson’s ‘The Composition of Foods’ integrated dataset from 2008 (FoodStandardsAgency, 2008). In recent years’ links between sodium intake and health have led the food industry to lower the salt content of processed foods and it was therefore possible that the sodium data used within the diet diary analysis could underestimate current intake.

In March 2015, an updated food composition dataset was published by the UK Department of Health (PublicHealthEngland, 2015) and has since been included as an update for DietPlan (DietPlan7). We therefore developed new dietary codes for the foods consumed in the diet diaries that included both the sulphur content as previously used, and the updated sodium values from the 2015 food composition dataset. The sodium intake as estimated by duplicate diet and DietPlan7 analysis was compared as before. Overall the data between the two methods was more comparable than with the older dataset for sodium intake, although there was still strong evidence of a positive bias (mean difference = 311 mg, *P* = 0.0041) and proportionately higher estimates (ratio of standard deviations, DP7/DD = 1.21, significance of linear trend, *P* = 0.0015). Summary plots for the comparison between DietPlan7 and duplicate diaries are provided as Supplementary figures.

High sodium intake has been linked to increased risk of high blood pressure and cardiovascular disease (He & MacGregor, 2015). Although the average intake of sodium in the UK is on a downward trend, it was still 76 per cent above the recommended maximum Reference Nutrient Intake of 2.4 g/day in 2014 and in this study was found to be 2.5 g/day on average by diet diary analysis (Table 3). As such there has been an active approach to the reduction of salt added to foods in recent years (Webster, Trieu, Dunford, & Hawkes, 2014) and it is therefore important to use up-to-date information on sodium content of foods in dietary analysis calculations. The change in salt added to processed foods is undergoing such a rapid change that even the updated databases in Dietplan7 (from 2015 data) may still be overestimating dietary sodium intake, which may explain the disparity between the diet record estimates and duplicate diet analysis.

### 3.7 Analysis of calcium, iron, magnesium, phosphorous, potassium and zinc in the diet using duplicate diet and diet diary analyses

Calcium, iron, magnesium, phosphorous, potassium and zinc intake were also estimated for the 18 diet diaries and compared to duplicate diet analysis. Table 3 shows there was little evidence of bias for calcium, iron and magnesium (*P* > 0.10), and weak evidence of bias for phosphorous (*P* = 0.063) and potassium (*P* = 0.092). For zinc, there was strong evidence of a positive bias (mean difference = 2.7 mg, P = 0.0007), and to overestimate (ratio of standard deviations DP6/DD = 1.52, significance of linear trend *P* = 0.008) by diet diary analysis. Compound figures showing Bland and Altman, scatter and boxplots for these minerals are provided as Supplementary figures.

Caution is also advised when using dietary analysis software for the estimation of other mineral intake such as iron. Many foods that may contain added minerals, e.g. breakfast cereals, food composition data is often the average data for a range of similar products and the actual amount of fortificant consumed can be brand dependant.

## 4 Conclusions

As expected, high levels of sulphur were obtained from consumption of meat and fish, mainly in the form of SAA. However, the analysis of sulphur content and partitioning in commonly consumed foods and vegetables undertaken in this study indicates as much as 89.5% of the total sulphur consumed in a typical diet may not be derived from SAA. Significant sulphur intake was also achieved from consumption of wheat and starchy foods, dairy and vegetables. If only SAA intake is monitored the significant contribution to total sulphur intake from these other foods will be disregarded. Alliaceous and cruciferous vegetables are shown to be an important source of sulphur in the diet.

The results of this study suggest there is no requirement for mass re-analysis of the composition of sulphur in foods and that existing data can be used with consumption data for estimation of sulphur intake. We also found evidence that dietary intake analysis overestimated the amount of sodium consumed which may be a consequence of the compositional data not keeping pace with the trend of manufacturing food with a reduced salt content. Total sulphur intake estimated from dietary intake diaries is comparable to that from duplicate diet analysis and can include a breakdown of the sulphur amino acid, sulphate and sulphur containing metabolites consumed.

## Conflicts of interest

None

## Authorship contributions

Joanne F Doleman: formulation of research question, preparation of the sulphur database, design of study, analysis of diet diaries, data analysis and writing of article.

Shikha Saha: analysis of food samples.

Katrijn Grisar: preparation of the sulphur database, analysis of diet diaries.

Lena Van Liedekerke: preparation of the sulphur database, analysis of diet diaries.

Henri S Tapp: data analysis.

Mark Roe: input to data for sulphur database, assistance in diet diary analysis, contribution to structure, content and writing of article.

Richard F Mithen: formulation of research question, design of study, contribution to structure, content and writing of article.

## Funding

The study was supported by a strategic programme grant to IFR from the UK Biotechnology and Biological Sciences Research Council [BB/J004545/1].

## Figures and Tables

**Supplementary Figure 1:**
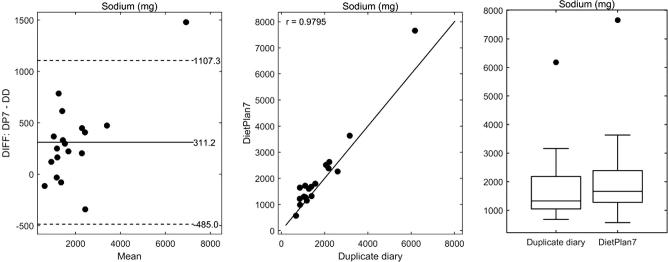
**Updated Sodium:** Sodium intake as estimated by updated databases in diet diary analysis data versus duplicate diet analysis. New Sodium data was obtained from the UK Department of Health updated food composition dataset (PublicHealthEngland, 2015) *Left panel*, Bland and Altman plot (Bland & Altman, 1986) of the differences, DietPlan7 – duplicate diary, against the mean of the two estimates. Also shown is the bias given as the mean difference and the 95% limits of agreement (mean difference ± 2 × standard deviation). *Central panel*, scatterplot of DietPlan7 estimates against duplicate diary values. Also shown is the Pearson correlation between the two methods and the line of unity. *Right panel*, boxplots summarising the distribution of the two estimates.

**Supplementary Figure 2:**
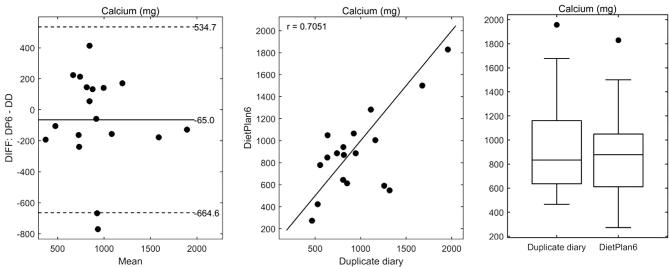
**Calcium:** Calcium intake estimates of 18 × 24-hour dietary intake diaries using DietPlan6 and duplicate diet analysis. *Left panel*, Bland and Altman plot, (Bland & Altman, 1986) of the differences, DietPlan6 – duplicate diary, against the mean of the two estimates. Also shown is the bias given as the mean difference and the 95% limits of agreement (mean difference ± 2 × standard deviation). *Central panel*, scatterplot of DietPlan6 estimates against duplicate diary values. Also shown is the Pearson correlation between the two methods and the line of unity. *Right panel*, boxplots summarising the distribution of the two estimates.

**Supplementary Figure 3:**
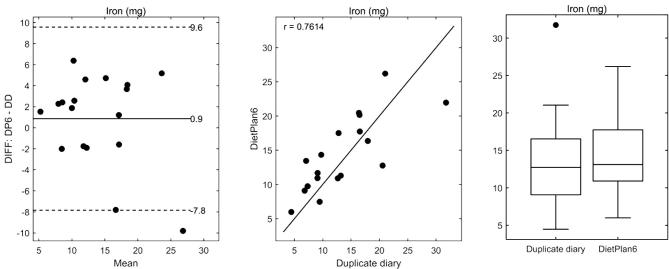
**Iron:** Iron intake estimates of 18 × 24-hour dietary intake diaries using DietPlan6 and duplicate diet analysis. *Left panel*, Bland and Altman plot (Bland & Altman, 1986) of the differences, DietPlan6 – duplicate diary, against the mean of the two estimates. Also shown is the bias given as the mean difference and the 95% limits of agreement (mean difference ± 2 × standard deviation). *Central panel*, scatterplot of DietPlan6 estimates against duplicate diary values. Also shown is the Pearson correlation between the two methods and the line of unity. *Right panel*, boxplots summarising the distribution of the two estimates.

**Supplementary Figure 4:**
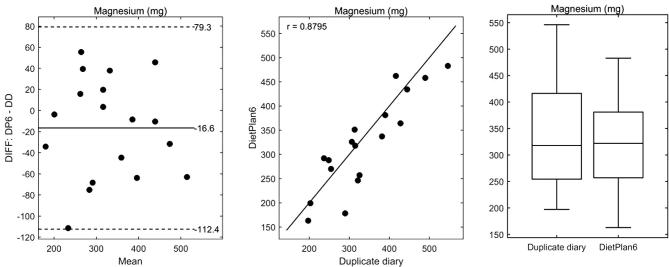
**Magnesium:** Magnesium intake estimates of 18 × 24-hour dietary intake diaries using DietPlan6 and duplicate diet analysis. *Left panel*, Bland and Altman plot (Bland & Altman, 1986) of the differences, DietPlan6 – duplicate diary, against the mean of the two estimates. Also shown is the bias given as the mean difference and the 95% limits of agreement (mean difference ± 2 × standard deviation). *Central panel*, scatterplot of DietPlan6 estimates against duplicate diary values. Also shown is the Pearson correlation between the two methods and the line of unity. *Right panel*, boxplots summarising the distribution of the two estimates.

**Supplementary Figure 5:**
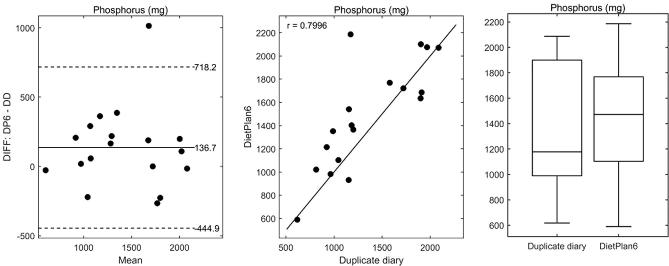
**Phosphorous:** Phosphorous intake estimates of 18 × 24-hour dietary intake diaries using DietPlan6 and duplicate diet analysis. *Left panel*, Bland and Altman plot (Bland & Altman, 1986) of the differences, DietPlan6 – duplicate diary, against the mean of the two estimates. Also shown is the bias given as the mean difference and the 95% limits of agreement (mean difference ± 2 × standard deviation). *Central panel*, scatterplot of DietPlan6 estimates against duplicate diary values. Also shown is the Pearson correlation between the two methods and the line of unity. *Right panel*, boxplots summarising the distribution of the two estimates.

**Supplementary Figure 6:**
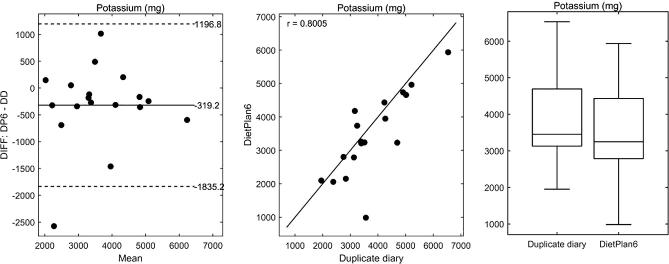
**Potassium:** Potassium intake estimates of 18 × 24-hour dietary intake diaries using DietPlan6 and duplicate diet analysis. *Left panel*, Bland and Altman plot (Bland & Altman, 1986) of the differences, DietPlan6 – duplicate diary, against the mean of the two estimates. Also shown is the bias given as the mean difference and the 95% limits of agreement (mean difference ± 2 × standard deviation). *Central panel*, scatterplot of DietPlan6 estimates against duplicate diary values. Also shown is the Pearson correlation between the two methods and the line of unity. *Right panel*, boxplots summarising the distribution of the two estimates.

**Supplementary Figure 7:**
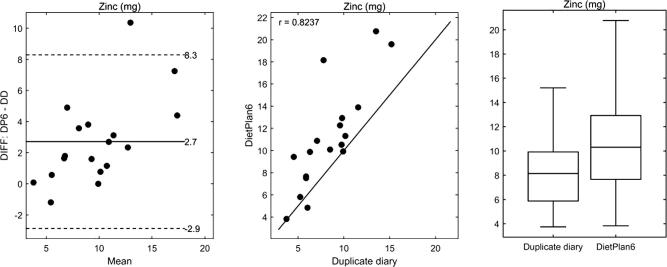
**Zinc**: Zinc intake estimates of 18 × 24-h dietary intake diaries using DietPlan6 and duplicate diet analysis. Left panel, Bland and Altman plot (Bland & Altman, 1986) of the differences, Dietplan6 – duplicate diary, against the mean of the two estimates. Also shown is the bias given as the mean difference and the 95% limits of agreement (mean difference ± 2 × standard deviation). Central panel, scatterplot of DietPlan6 estimates against duplicate diary values. Also shown is the Pearson correlation between the two methods and the line of unity. Right panel, boxplots summarising the distribution of the two estimates.
